# The combination of long-acting injectable antipsychotics, a new key in resistant schizophrenia

**DOI:** 10.1192/j.eurpsy.2022.1875

**Published:** 2022-09-01

**Authors:** M.F. Tascón Guerra, A. Osca Oliver, M.V. López Rodrigo, M. Palomo Monge, M. Pérez Fominaya

**Affiliations:** 1 Hospital Nuestra Señora del Prado, Psiquiatria, Talavera de la Reina, Spain; 2 Hospital Nuestra Señora del Prado, Psiquiatría, Talavera de la Reina, Spain

**Keywords:** paliperidone, Aripiprazol, Long-acting injectable antipsychotics, schizophrénia

## Abstract

**Introduction:**

Schizophrenia is a chronic disease that requires lifelong medical care and supervision. There is a high rate of relapse, mostly caused by poor adherence to oral antipsychotics. Long-acting injectable (LAI) antipsychotics have proved effective in schizophrenia and other severe psychotic disorders due to the stable blood levels, leading to a reduction of the risk of relapse. LAIs are associated with better functioning, quality of life, and patient satisfaction. In Treatment-resistant schizophrenia the combination of antipsychotics is a common practice. Nevertheless, the combination of two different long-acting injectable antipsychotics is not frequent.

**Objectives:**

A case of a 34-year-old man is presented, previously diagnosed of Schizophrenia, with highly disabling chronic positive symptoms. With no in-sight and no will in receiving treatment. Has been stable for a year while being in treatment with paliperidone 525mg LAI/ 10 weeks, and aripiprazole 400mg LAI/28 days.

**Methods:**

The patient was closely observed and given oral paliperidone, after 5 days long-acting paliperidone was introduced. He was discharged with mild improvement of his psychiatric symptoms. While being in treatment with Paliperidone 525mg, he kept vivid delusions and hallucinations. The patient still refused to take any oral medications. Long-acting aripiprazole 300mg was added to the treatment.

**Results:**

He showed clinical improvement after a month. He has been stabilized for one year.

**Conclusions:**

Treating resistant schizophrenia is among the most challenging clinical endeavors. A very helpful approach to improve adherence in schizophrenia is the use of long-acting injectable (LAI) antipsychotics. A major effort on scientific research of combination of LAI is needed.

**Disclosure:**

No significant relationships.

